# *Polygonatum sibiricum* Polysaccharides Alleviate Simulated Weightlessness-Induced Cognitive Impairment by Gut Microbiota Modulation and Suppression of NLRP3/NF-κB Pathways

**DOI:** 10.3390/nu17193157

**Published:** 2025-10-05

**Authors:** Fang Chen, Muhammad Noman Khan, Mengzhou Xie, Yiwen Zhang, Liang Li, Ahsana Dar Farooq, Jixian Liu, Qinghu He, Xinmin Liu, Ning Jiang

**Affiliations:** 1 Research Center for Pharmacology and Toxicology, Institute of Medicinal Plant Development (IMPLAD), Chinese Academy of Medical Sciences and Peking Union Medical College, Beijing 100193, China; t15115195367@163.com (F.C.); nomankhan.phd@hotmail.com (M.N.K.); ywzhang0406@163.com (Y.Z.); 2 Sino-Pakistan Center on Traditional Chinese Medicine, Hunan University of Medicine, Huaihua 418000, China; hqh19651111@163.com; 3 School of Traditional Chinese Medicine, Hunan University of Chinese Medicine, Changsha 410208, China; xiemz@163.com (M.X.); superliliang@126.com (L.L.); 4 Faculty of Eastern Medicine, Hamdard University, Karachi 74600, Pakistan; ahsanadar@hotmail.com; 5 Institute of Drug Discovery Technology, Ningbo University, Ningbo 315211, China; liujixian_caresse@163.com

**Keywords:** *Polygonatum sibiricum* polysaccharides, cognitive impairment, microbiota–gut–brain, neuroinflammation

## Abstract

**Background/Objectives**: *Polygonatum sibiricum* (PS), possessing both medicinal and edible dual functions, boasts a long history of application in Chinese traditional practices. As a component of its effectiveness, *Polygonatum sibiricum* polysaccharides (PSPs) have been reported to exert neuroprotective effects. However, the protective effects of PS on the cognitive deficits induced by simulated weightlessness remain unclear. This study evaluated the therapeutic potential of PSPs to counteract the cognitive deficits induced by simulated weightlessness using the Hindlimb Unloading (HU) method. **Methods**: Mice were subjected to HU to establish cognitive impairment, and PSP was administered for four weeks. The Morris water maze test (MWMT) and passive avoidance test (PAT) were used to evaluate the cognitive abilities of mice, followed by an analysis of molecular mechanisms. **Results**: PSP treatment increased learning and memory in mice. PSP treatment partially restored gut microbial diversity and composition towards beneficial taxa, including *Lactobacillus* and *Firmicutes*, while inhibiting proinflammatory genera, including *Alistipes* and *Proteus*. At the same time, PSP upregulated Claudin-5 and Zonula Occludens-1 (ZO-1) levels in the colon, suggesting improved intestinal barrier integrity, and decreased neuroinflammatory response by inhibiting NLRP3 inflammasome activation and NF-κB phosphorylation in the hippocampus. It also modulated neurotransmitter homeostasis along the microbiota–gut–brain (MGB) axis by increasing the levels of gamma-aminobutyric acid (GABA) and serotonin (5-HT) while reducing the levels of excitotoxic metabolites, including Glutamate (Glu) and 3-hydroxykynurenine (3-HK). **Conclusions**: These results indicate that PSP may have beneficial effects on HU-induced cognitive impairment by regulating gut microbiota, enhancing barrier function, suppressing neuroimmune signaling, and restoring neurotransmitter balance.

## 1. Introduction

Throughout deep space missions, astronauts encounter a multitude of stressors inherent to the space environment, such as microgravity. These environmental factors not only affect their physiological homeostasis but may also induce neurocognitive disturbances, potentially compromising crew safety and mission success [1,2]. This aerospace environment-induced condition is a functional impairment without a clear lesion site or key target. Although NASA, with other partner nations, has developed a variety of countermeasures, including training, exercises, and pharmacological treatments, these interventions often yield limited neuroprotection in weightlessness [3]. Therefore, preventing or mitigating learning and memory impairments in space-weightless environments remains a critical challenge.

The intestinal microbiota is a complex microbial ecosystem that is relatively stable under normal circumstances and maintains a harmonious symbiotic relationship with the body [4]. Studies reported that prolonged exposure to weightless environments can cause imbalances in microbial ecosystems, and these changes contribute to cognitive impairment and neuroinflammation [5,6,7]. The disruption of gut microbiota does not only affect the human immune system, as evidenced by the elevated levels of inflammatory cytokines and altered levels of neurotransmitters such as gamma-aminobutyric acid (GABA) and glutamic (Glu), as well as tryptophan and its metabolites. It also stimulates the occurrence of intestinal and peripheral inflammation, leading to the breakdown of the blood–brain barrier and ultimately impairing cognitive function. This two-way relationship between the gut microbiota and cognitive impairment, known as the microbiota–gut–brain (MGB) axis, is closely associated with cognitive function.

*Polygonatum sibiricum* (PS) is a traditional Chinese medicine with neuroprotective, anti-aging, liver-protecting, and immune-boosting properties [8,9]. *Polygonatum sibiricum* polysaccharides (PSPs), a bioactive compound extracted from PS, have antioxidant, anti-inflammatory, neuroprotective, and immune-boosting properties [10]. Previous studies have explored the neuroprotective effects of PSP in Alzheimer’s disease, chronic restraint stress, and sleep deprivation [11,12,13]. However, its impact on the cognitive impairment caused by simulated weightlessness during spaceflight has seldom been explored.

In this study, we investigated the effects and possible mechanisms by which PSP improves cognitive impairment induced by a Hindlimb Unloading (HU) model and its potential application value in aerospace medicine.

## 2. Materials and Methods

### 2.1. Drugs

PSP (Batch Number: S27804, purity > 70%) was obtained from Yuanye Bio-Technology (Shanghai, China); Huperzine A was procured from Sigma-Aldrich (St. Louis, MO, USA); Arginase-l (Arg1) ELISA Kit (MU30384) was obtained from Bioswamp Biological (Wuhan, China).

### 2.2. Animals and Treatments

**Figure 1:**
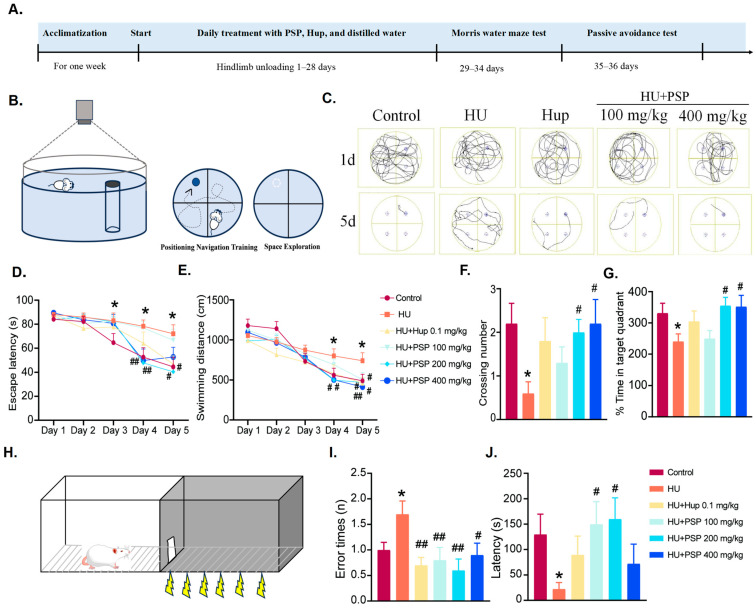
Effects of PSP on MWMT and PAT in HU mice. (**A**) Flow diagram of experiment. (**B**) Schematic diagrams of MWMT. (**C**) Track plots of mice in MWMT. (**D**,**E**). Escape latency and swimming distance in acquisition of MWMT. (**F**,**G**) Crossing numbers and percentage of swimming distance in target quadrant in probe trial in MWMT. (**H**) Schematic diagram in PAT. (**I**) Error times in PAT. (**J**) Escape latency in PAT (n = 10, mean ± SEM). * *p* < 0.05 vs. control group; ^#^ *p* < 0.05, ^##^ *p* < 0.01, vs. HU group.

Male ICR mice (4–6 weeks) were obtained from Viton Lihua Laboratory. The animals were acclimated to the experimental animal facility, where the temperatures were regulated, and a consistent light–dark cycle was maintained. All experiments obtained approval from the Institutional Animal Care and Use Committee of the Institute of Medicinal Plant Development, Chinese Academy of Medical Sciences (Approval Number 2023-0008). Mice were randomly allocated to six groups: (1) control group (control, distilled water), (2) Hindlimb Unloading group (HU, distilled water); (3) Huperzine A treatment group (Hup, 0.1 mg/kg); (4) PSP treatment group (100 mg/kg); (5) PSP treatment group (200 mg/kg); and (6) PSP treatment group (400 mg/kg). All the drugs were administered via the intragastric (i.g.) route. PSP and Hup doses were chosen based on our previous studies [11,14]. Using the standard body surface area conversion formula, our highest dose (400 mg/kg in mice) corresponds to ~32 mg/kg in humans, which falls within a feasible range for nutritional supplements. The experimental schedule is shown in Figure 1A.

### 2.3. Hindlimb Unloading (HU) Procedures

The model animals were suspended individually in a transparent plexiglass cage (26 cm × 26 cm × 30 cm) for 28 consecutive days using a method similar to that previously used for mice [15]. Briefly, the mice’s tails were firmly fastened using medical adhesive tape and hung from stainless steel chains attached to the top of the cage. Height adjustments were ensured so that each animal’s hindlimbs were elevated and the head was tilted 25–30° for a long time.

### 2.4. Morris Water Maze Test

As described in previous studies [16], the apparatus used in this experiment is a large circular pool with a diameter of 180 cm, which was rendered opaque by mixing in black material. The MWM test spans 6 consecutive days, with the first 5 days being the hidden platform phase. During this phase (the acquisition phase), escape latency is employed as an indicator to assess spatial memory ability in mice. On day 6, the platform was removed, and each mouse was allowed to explore freely for 90 s (the probe trial phase). The computerized tracking system recorded the number of platform crossings and other indicators. In the MWM test, shorter latency to locate the hidden platform, more platform crossings during the probe trial, and enhanced preference for the target quadrant collectively exhibit superior spatial learning and memory capabilities.

### 2.5. Passive Avoidance Test

During the acquisition phase, mice were gently placed inside the illuminated chamber to adapt for 3 min. Subsequently, the current was immediately turned on, and the mice were subjected to continuous electric shocks for 5 min. In the retention phase, the mice were placed directly into the illuminated chamber, and electric shocks were administered immediately, with the duration kept consistent with that of the acquisition phase.

### 2.6. Blood and Tissue Sample Collection

After the conclusion of behavioral tests, all mice were anesthetized with pentobarbital (intraperitoneal, 70 mg/kg). The blood was collected from the orbital venous plexus into 1.5 mL Eppendorf tubes. Mice were then immediately sacrificed on ice, and whole brains were rapidly isolated. The brain tissues, including the hippocampus and cortex, were dissected on ice and immediately stored at −80 °C. Colon tissue was also excised, washed with cold PBS, and stored in the same conditions.

### 2.7. 16s rRNA Gene Sequencing

16s rRNA amplification and sequencing were carried out as described before [17]. The HiPure Fecal DNA Kit (Magen, Guangzhou, China) was used to extract total genomic DNA from samples according to the manufacturer’s protocol. After extracting genomic DNA from the samples, the bacterial 16sV3–V4 rDNA region was amplified. Raw data were acquired using the Illumina MiSeq sequencing platform. A HiSeq2500 PE250 from GeneDenovo (Guangzhou, China) was used for analysis. For β-diversity comparisons, we used Principal Coordinate Analysis (PCoA) based on Bray–Curtis distances, and a PERMANOVA (999 permutations) was performed to assess statistical significance. In differential taxon analysis, the LefSe method was applied, which incorporates the Kruskal–Wallis test and LDA score filtering.

### 2.8. Enzyme-Linked Immunosorbent Assay

Commercial ELISA kits were utilized to assess the serum levels of Arg1, following the procedures specified by the manufacturers.

### 2.9. Real-Time Quantitative PCR

The protocol for total RNA extraction from the cortex, hippocampus, and colon was similar to the methods described in our previous study [18]. Briefly, total RNA extraction was performed with TRIzon reagent (CWBIO, Taizhou, China), followed by concentration determination using the Nanodrop 2000 (Thermo Fisher Scientific, Waltham, MA, USA). The cDNA synthesis reaction was initiated with 20 μL reaction mix, which was then incubated under defined thermal cycling conditions to eliminate genomic DNA contamination and reverse-transcribe mRNA into complementary DNA. Equal volumes of the synthesized cDNA were subsequently used as templates in the Bio-Rad CFX96 Real-Time PCR Detection System for quantitative reverse transcription PCR (qRT-PCR) analysis. Relative gene expression was calculated using the 2^−ΔΔCt^ method. The sequences of qPCR primers are described in Supplementary Table S1, which were synthesized by Sango Biotech (Ningbo, China).

### 2.10. Neurotransmitter Detection

The experimental procedures adhered to the validated sample treatment protocol described by our group with minor modifications [19]. Briefly, ultra-fast liquid chromatography (UFLC) and the QTRAP 5500 mass spectrometer QTRAP 5500 mass spectrometer (LC-20AT, Shimadzu Corporation, Kyoto, Japan) were used to determine neurotransmitter levels in the hippocampus, cortex, serum, and colon. The RESTEK ultra water C18 column was applied in the UFLC system for metabolite separation.

### 2.11. Western Blot Assay

The method used for Western blot assay was in accordance with previous research [20]. Colon and hippocampal tissues were lysed in RIPA buffer (Beyotime, P0023A), containing protease and phosphatase inhibitors (Solarbio, P1261, Beijing, China). Protein concentrations were determined using the BCA Kit (Beyotime, Nanjing, China). Equivalent quantities of protein samples were subjected to SDS-PAGE, after which they were transferred onto NC membranes. Subsequently, the membranes were blocked with 5% skimmed milk and incubated overnight at 4 °C with respective primary antibodies as follows, following incubation with HRP-conjugated secondary antibodies: NLRP3, 1:1000, CST; NF-κB, 1:1000, CST; Phospho-NF-κB, 1:1000, CST; IL-1β, 1:1000, ImmunoWay; caspase-1, 1:1000, CST; ASC, 1:1000, CST; ZO-1, 1:1000, Proteintech; Claudin-5, 1:1000, Proteintech; and GAPDH, 1:2500. Finally, protein bands were visualized using the Beyo ECL Moon kit (Beyotime, China). A densitometric analysis of protein bands was performed using ImageJ software (Version 1.53k, National Institute of Health, Bethesda, MD, USA).

### 2.12. Statistical Analysis

All statistical analyses were performed using SPSS (ver. 26.0), and data were expressed as the mean ± SEM. For the differences among data values that conform to a normal distribution, we employed a one-way ANOVA or repeated-measures two-way ANOVA, and the LSD was used for the post-test. Otherwise, the Mann–Whitney U test was used. *p* < 0.05 was defined as statistically significant. Graphical representations were created through GraphPad Prism 8.0 software.

## 3. Results

### 3.1. Effects of PSP on Morris Water Maze Test in Mice with HU

During the acquisition phase, as training progressed, all groups of mice exhibited a progressive reduction in escape latency, as illustrated in Figure 1D. From day 3 to day 5, the mice in the HU group showed significantly extended escape latencies (*p* = 0.045; *p* = 0.017; and *p* = 0.024, respectively), indicating impaired memory during long-term modeling. Meanwhile, the test revealed that PSP 200 and 400 mg/kg significantly reduced the escape latency on day 4 (*p* < 0.01). Hup and PSP 200 mg/kg treatment reduced the escape latency on day 5 (*p* < 0.05). As shown in Figure 1E, HU-induced mice exhibited a significantly longer swimming distance than did the control group on days 4 to 5 (*p* = 0.048; *p* = 0.029). After treatment with PSP 200 and 400 mg/kg, the swimming distance was significantly lower on day 4 (*p* < 0.05). Notably, Hup and all PSP doses reversed adverse changes on day 5 (*p* < 0.05).

Furthermore, in the subsequent space exploration session, the model group showed impaired memory (*p* = 0.011; *p* = 0.035, Figure 1F,G). This impairment was ameliorated by PSP (200 and 400 mg/kg) treatment. Meanwhile, the movement trajectory map of HU group mice exhibited a lack of purposefulness (Figure 1C).

### 3.2. Effects of PSP on Passive Avoidance Test in Mice with HU

As shown in Figure 1J, HU significantly shortened latency (*p* = 0.049). PSP (100 and 200 mg/kg) administration significantly reversed it (*p* < 0.05). In addition, the number of errors was significantly higher in the model group of mice (Figure 1I, *p* = 0.026). Both Hup and all PSP doses reduced errors significantly (*p* < 0.05).

### 3.3. Shift in Gut Microbiome in Mice Treated with PSP

**Figure 2:**
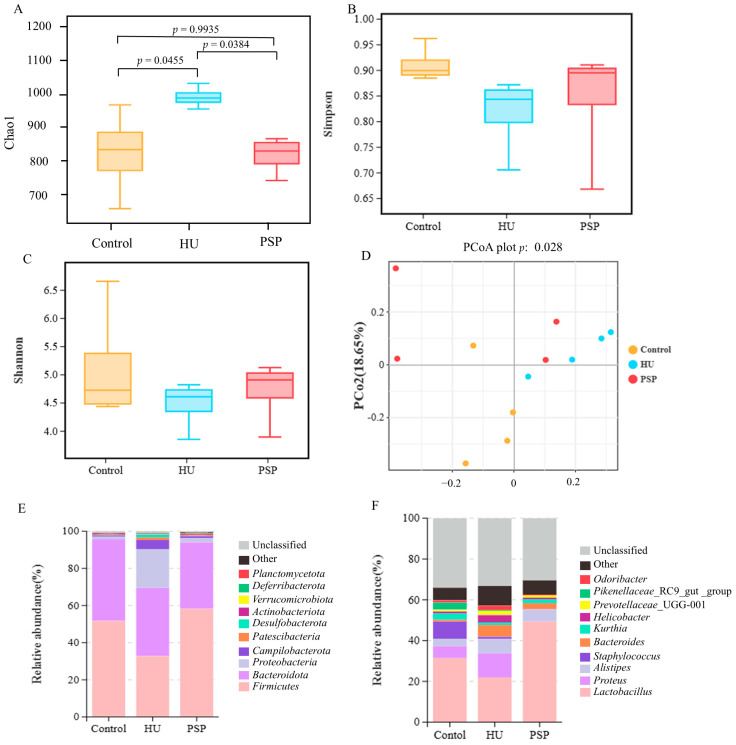
Shift in gut microbiome in mice treated with PSP. (**A**) Chao1 index. (**B**) Simpson index. (**C**) Shannon index. (**D**) PCoA. (**E**,**F**) Abundance histogram of intestinal microflora in mice at phylum and genus levels. n = 4.

When assessing the diversity of the gut microbiome, alpha diversity serves as an indicator that reflects both the abundance and variety of species present within a given microbial community. The total number of species in each community was estimated using the Chao1 index. As depicted in Figure 2A, the HU group exhibited more unobserved species than the control group. This means that HU modeling induced more unknown changes within the intestines (*p* = 0.0455). Notably, the administration of PSP successfully mitigated this irregular microbial alteration (*p* = 0.0384). The Simpson and Shannon indices of the gut microbiota showed a constant increasing trend in the PSP group (Figure 2B,C). Beta diversity analysis was employed to evaluate the differences in intestinal microorganisms among three groups of mice. The Principal Coordinate Analysis (P-CoA) is shown in Figure 2D, and the results showed significant differences (*p* < 0.05). The dilution curves of each sample tend to be flat, indicating that the number of OTUs contained in the samples tends to be stable (Supplementary Figure S1). The amount of sequencing data was reasonable. There were 232 OTUs specific to the HU group and 132 OTUs unique to the PSP group, indicating that the HU model brought about changes in the quantity of OTUs within the gut microbiota of mice. Moreover, PSP intervention played a role in partially mitigating these abnormal OTU fluctuations (Supplementary Figure S2).

As shown in Figure 2E, the abundance of the top 10 taxa was evaluated across different groups. *Firmicutes*, *Bacteroidota*, and *Proteobacteria* were the three predominant phyla among intestinal microorganisms. HU decreased the *Firmicutes*/*Bacteroidota* (F/B) ratio in the HU group (*p* < 0.05). PSP treatment significantly reversed these changes (Supplementary Figure S3). PSP also reversed the increases in the abundance of *Proteobacteria* in HU-treated mice. At the genus level, HU led to an increase in the relative abundance of *Proteus*, *Alistipes*, and *Bacteroides* and a decrease in *Lactobacillus*. However, this phenomenon was reversed after PSP treatment (Figure 2F).

Furthermore, we used Lef-Se to analyze the significantly different species between groups (a logarithmic LDA score cutoff of 3). The results are shown in Supplementary Figure S4. A total of four biomarkers were detected in the control group, six taxon biomarkers were detected in the HU group, and three taxon biomarkers were detected in the PSP group. The taxa with the most significant differences in abundance were *Erysipelotrichales*, *Sphingomonas glacialis*, *Myxococcaceae*, and *Marine Benthic* Group D and DHVEG 1 for the control group; *Bacteroides acidifaciens*, *Helicobacter typhlonius*, *Oscillos pirales*, *Streptococcaceae*, *Lactococcus*, *Ruminococcaceae*, *Prevotellaceae* NK3B31 group, and *Spirochaeta* for the HU group; and *Corynebacteriales*, *Corynebacterium*, and *Corynebacteriaceae* for the PSP group. KEGG metabolic pathway analysis based on functional annotation (Supplementary Figure S4) revealed significant compositional differences in microbial functional genes across sample groups. The differentially enriched microbial communities encompass multiple functional categories, including metabolism, genetic information processing, cellular processes and signaling, and human diseases, indicating their potential roles in host–microbe interactions and disease pathogenesis. However, the underlying mechanisms driving these microbiota-induced metabolic alterations require further investigation.

### 3.4. Effect of PSP on mRNA Expression of Inflammatory Cytokines in Mice with HU

**Figure 3:**
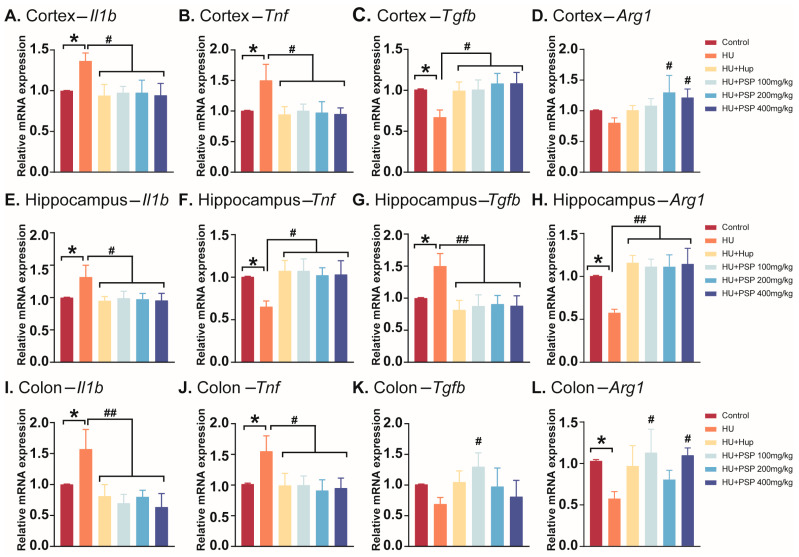
Effects of PSP on mRNA expression of inflammatory factors in brain tissues and colon in HU mice. (**A**–**D**) Levels of *Il1b*, *Tnf*, *Tgfb*, and *Arg1* in cortex. (**E**–**H**) Levels of *Il1b*, *Tnf*, and *Arg1* in hippocampus. (**I**–**L**) Levels of *Il1b*, *Tnf*, *Tgfb*, and *Arg1* in cortex (n = 6, mean ± SEM). * *p* < 0.05 vs. control group. ^#^ *p* < 0.05, ^##^ *p* < 0.01, vs. HU group.

As shown in Figure 3A–D, HU led to a significant increase in the relative mRNA expression levels of *Il1b* and *Tnf* (*p* = 0.034; *p* = 0.026) and the depletion of the relative mRNA expression levels of *Tgfb* in the cortex (*p* = 0.049), but there was no significant change in *Arg1*. Hup treatment significantly decreased the mRNA expression levels of *Il1b* and *Tnf* and increased the gene expression of *Tgfb* (*p* < 0.05). All PSP doses decreased the mRNA expression levels of *Il1b* and *Tnf* (*p* < 0.05) and significantly increased *Tgfb* expression (*p* < 0.05). PSP 200 and 400 mg/kg doses elevated *Arg1* expression (*p* < 0.05).

Similarly, in the hippocampus region (Figure 3E–H), HU group mice showed a significant increase in the relative mRNA expression levels of *Il1b* and *Tnf* (*p* = 0.032; *p* = 0.032), and the relative mRNA expression levels of *Tgfb* and *Arg1* were significantly decreased (*p* = 0.044; *p* = 0.024). Treatment with Hup and all doses of PSP significantly decreased the mRNA expression levels of *Il1b* and *Tnf* (*p* < 0.05). Hup and all doses of PSP administration increased gene expression in *Tgfb* and *Arg1* (*p* < 0.01).

Furthermore, it was found that changes in inflammatory cytokine mRNA expression in the colon were essentially similar to those in the hippocampus, as shown in Figure 3I–L. The results demonstrated that HU markedly elevated the relative mRNA expression levels of *Il1b* and *Tnf* and reduced those of *Arg1* (*p* = 0.047; *p* = 0.043; *p* = 0.050) but not *Tgfb*. Hup and all doses of PSP significantly reduced the relative mRNA expression levels of not only *Il1b* but also *Tnf* (*p* < 0.05). Additionally, PSP 100 mg/kg doses increased the mRNA expression level of *Tgfb* (*p <* 0.05), while PSP treatment (100 mg/kg and 400 mg/kg) elevated the mRNA expression level of *Arg1* (*p* < 0.05).

### 3.5. Effect of PSP on Neurotransmitter Levels in Mice with HU

**Figure 4:**
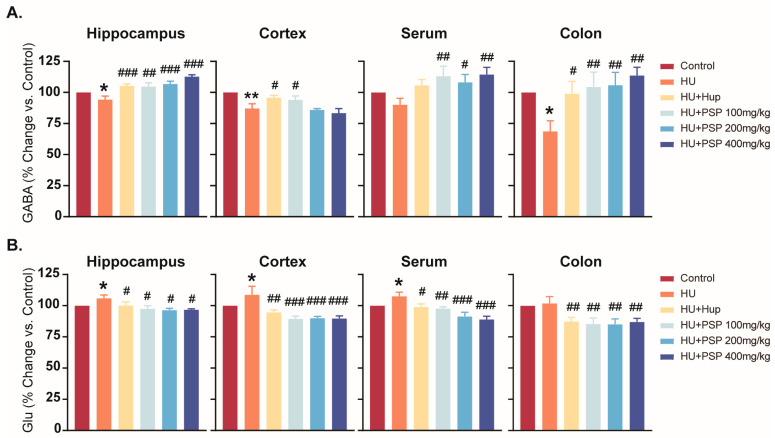
Effects of PSP on neurotransmitter levels in brain tissues, serum, and colon regions in HU mice. (**A**) Levels of GABA in hippocampus, cortex, serum, and colon (% Changes vs. Control). (**B**) Levels of Glu in hippocampus, cortex, serum, and colon (% Changes vs. Control) (n = 8, mean ± SEM). * *p* < 0.05, ** *p* < 0.01, vs. control group. ^#^ *p* < 0.05, ^##^ *p* < 0.01, ^###^ *p* < 0.001, vs. HU group.

HU group mice showed a significant decrease in hippocampus, cortex, serum, and colon GABA levels compared to controls (*p* < 0.05, Figure 4). Treatment with Hup significantly reversed the abnormal changes in GABA in brain tissues (*p* < 0.05). PSP treatment increased GABA levels in the hippocampus (*p* < 0.05). Furthermore, PSP at 100 mg/kg and 400 mg/kg significantly increased GABA levels in the cortex and serum (*p* < 0.05), and both 100 mg/kg and 400 mg/kg significantly elevated GABA levels in the colon (*p* < 0.05).

HU led to a significant increase in the levels of Glu in the hippocampus, cortex, and serum (*p* < 0.05) but not in the colon. Positive control drug treatment significantly decreased Glu levels in the hippocampus, cortex, and serum (*p* < 0.05). PSP at all doses reduced Glu levels in all regions (*p* < 0.05).

The levels of E and NE in brain tissues in the HU group were significantly reduced (*p* < 0.05). Hup (10 mg/kg) treatment significantly increased E expression in brain tissues and NE in the cortex (*p* < 0.05). PSP intervention reversed the aberrant changes in E and NE levels caused by HU (*p* < 0.05). HU also reduced E levels in the colon (*p* < 0.05) while increasing NE levels in the serum and colon (*p* < 0.05). Hup (10 mg/kg) treatment significantly increased E expression in the serum and colon (*p* < 0.05). PSP at 100 mg/kg and 200 mg/kg doses significantly reversed abnormal changes in E levels in the colon (*p* < 0.05). All PSP doses and PSP 100 mg/kg doses reduced NE levels in the serum and colon (*p* < 0.05, Supplementary Figure S5).

### 3.6. Effect of PSP on TRP Metabolites in Mice with HU

**Figure 5:**
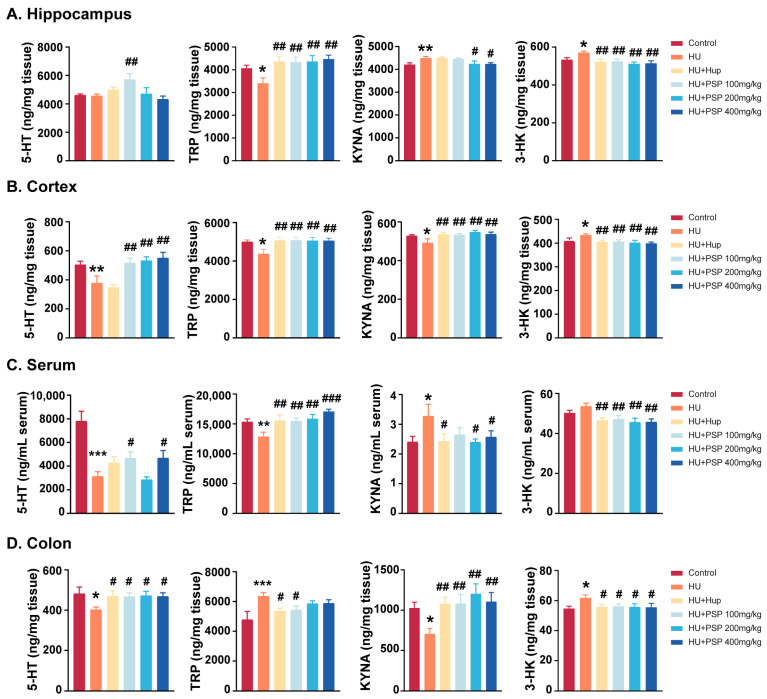
Effects of PSP on TRP metabolites in hippocampus, cortex, serum, and colon of mice with HU. (**A**–**D**) Levels of 5-HT, TRP, KYNA, and 3-HK in hippocampus, cortex, serum, and colon (n = 8, mean ± SEM). * *p* < 0.05, ** *p* < 0.01, *** *p* < 0.001, vs. control group. ^#^ *p* < 0.05, ^##^ *p* < 0.01, ^###^ *p* < 0.001, vs. HU group.

To evaluate the disparities in TRP metabolism, the quantities of TRP metabolites were measured in four distinct segments (Figure 5).

HU reduced TRP levels in the brain tissues of mice (*p* = 0.038; *p* = 0.015). However, it increased TRP levels in the colon (*p* < 0.001). In the circulatory system, a notable decline in serum TRP levels was observed (*p* < 0.01). This was similar to the situation for the brain but opposite to that for the colon. Hup (10 mg/kg) and PSP treatment significantly increased TRP expression levels, except for in the colon (*p* < 0.01). Hup and PSP 100 mg/kg markedly decreased TRP levels in the colon (*p* < 0.01).

KYNA levels were generally reduced in brain tissues and increased in the peripheral tissues (serum and colon) (*p* < 0.01). After treatment with Hup and all doses of PSP, KYNA levels in the cortex and hippocampus were increased (*p* < 0.01). The administration of Hup and PSP (200, 400 mg/kg) significantly reduced KYNA levels in the serum (*p* < 0.05), and both PSP doses (200, 400 mg/kg) significantly decreased KYNA expression in the colon (*p* < 0.05).

Simultaneously, a significant elevation was observed in neurotoxic 3-HK levels within brain regions (*p* = 0.020; *p* = 0.038). The level of 3-HK in the colon was also increased notably (*p* = 0.010) but not in the serum. The HU-induced increase in 3-HK levels was significantly inhibited after treatment with Hup and all doses of PSP (*p* < 0.05).

As expected, HU significantly decreased the cortex, serum, and colon levels of 5-HT (*p* < 0.05) but not in the hippocampus. However, PSP 100 mg/kg significantly elevated 5-HT levels in the hippocampus (*p* < 0.01). All doses of PSP increased 5-HT levels in the cortex (*p* < 0.01). Administration with Hup and all doses of PSP markedly increased 5-HT levels in the colon (*p* < 0.05). The reduced levels of 5-HT in the serum were significantly reversed following treatment with PSP 100 and 400 mg/kg (*p* < 0.05).

### 3.7. Effects of PSP on Intestinal Barrier Function of Mice with HU

**Figure 6:**
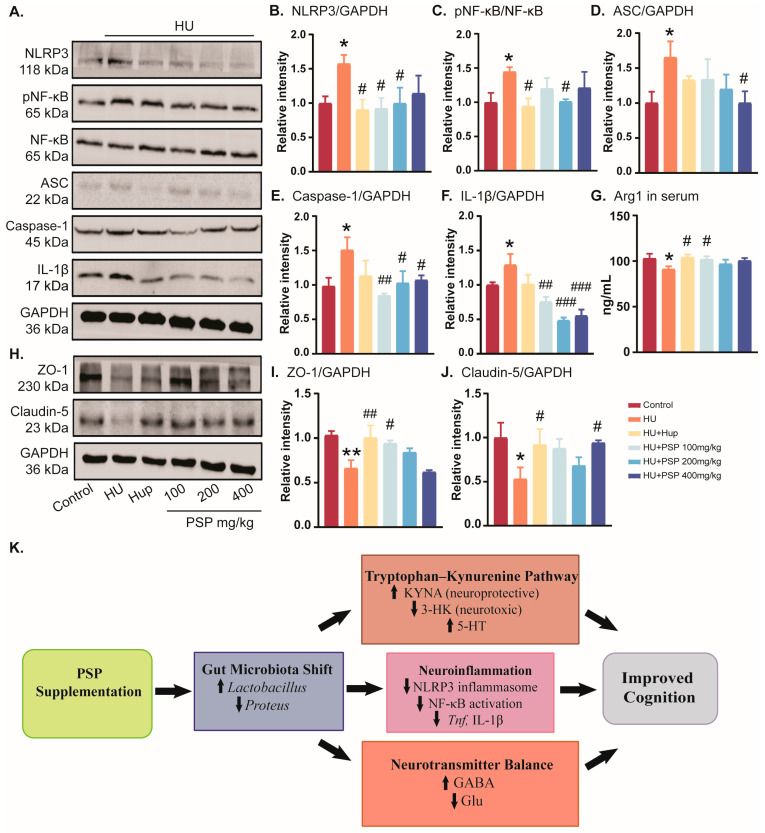
Effects of PSP on inflammatory response and NLRP3/NF-κB pathways and intestinal barrier function in mice with HU. (**A**) Protein bands in hippocampus tissues. (**B**–**F**) Levels of NLRP3, *p*-NF-κB/NF-κB, ASC, caspase-1, IL-1β, and GAPDH in hippocampus. (**G**) Levels of Arg1 in serum. (**H**) Protein bands in colon. (**I**,**J**) Levels of ZO-1, Claudin-5, and GAPDH in colon. (**K**) Schematic flow chart of proposed mechanism of PSP in HU-induced cognitive impairment. ↑ = increase, ↓ = decrease. (n = 4 in hippocampus and colon; n = 10 in serum, mean ± SEM). * *p* < 0.05, ** *p* < 0.01, vs. control group. ^#^ *p* < 0.05, ^##^ *p* < 0.01, ^###^ *p* < 0.001, vs. HU group.

HU suppressed the expression of Claudin-5 and ZO-1 in the colon (Figure 6I,J, *p* = 0.018; *p* = 0.002). By contrast, Claudin-5 and ZO-1 levels significantly increased after treatment with Hup A and PSP (100 and 400 mg/kg) (*p* < 0.05). This indicates that PSP can improve the dysfunction of the intestinal barrier in HU mice by boosting the synthesis of relevant tight junction proteins.

### 3.8. Effects of PSP on Serum Inflammatory Response and NLRP3/NF-κB Pathways in Hippocampus of Mice with HU

HU exposure significantly elevated NLRP3 and *p*-NF-κB/NF-κB expression in the hippocampus (*p* = 0.035; *p* = 0.032, Figure 6B,C). Hup treatment reduced NLRP3 and *p*-NF-κB/NF-κB levels (*p* = 016; *p* = 0.018). PSP (100–200 mg/kg) reversed the abnormal NLPR3 level (*p* = 0.018; *p* = 0.034). Furthermore, PSP (200 mg/kg) intervention notably inhibited *p*-NF-κB/NF-κB expression (*p* = 0.037). In HU mice, the levels of ASC, caspase-1, and IL-1β increased significantly (*p* = 0.030; *p* = 0.020; *p* = 0.049, Figure 6D–F). All doses of PSP suppressed caspase-1 and IL-1β increases (*p* < 0.05), while PSP 400 mg/kg reduced ASC expression (*p* < 0.05).

The expression level of anti-inflammatory factor Arg1 in serum was significantly increased in the HU group (*p* = 0.026). Hup and PSP 100 mg/kg administration significantly reversed the decrease in Arg1 (*p* = 0.014; *p* = 0.045, Figure 6G).

## 4. Discussion

In this study, PSP effectively ameliorates cognitive deficits in mice induced by HU. This neuroprotective effect appears to be associated with the modulation of gut microbiota and the suppression of neuroinflammatory signaling pathways, specifically through the inhibition of NLRP3 inflammasome activation, and regulates neurotransmitter homeostasis. These results suggest the potential of PSP as a preventive measure against the cognitive decline associated with spaceflight, a critical challenge in aerospace medicine.

Cognitive deficits were successfully induced in mice in the HU model, as demonstrated by their poor performance in MWMT and PAT, which is consistent with previous studies of microgravity analog-induced cognitive impairments [21]. These behavioral changes are the result of hippocampus-dependent spatial learning and memory impairments that have been reported in rodents exposed to microgravity analogs [22]. Prolonged escape latency, reduced platform crossings, and increased passive avoidance errors collectively support the idea that cognitive function is disrupted by simulated weightlessness, consistent with previous reports [23]. Meanwhile, these deficits were significantly reversed in mice treated with PSP, particularly at dosses of 200 and 400 mg/kg, which improved escape latency and memory retention. These results are consistent with previous studies that showed the neuroprotective effects of PSP in animal models of Alzheimer’s disease and stress-induced cognitive impairment [11,24,25]. PSP also demonstrated similar efficacy with Huperzine A, a known acetylcholinesterase inhibitor, which indicates its therapeutic potential in cognitive dysfunctions associated with stressors encountered during spaceflight [26].

Huperzine A is a clinically used acetylcholinesterase inhibitor with documented neuroprotective and cognition-enhancing effects in rodent models and in Alzheimer’s patients [27,28]. Its mechanism of action complements PSP’s multi-target bioactivity, making it an appropriate benchmark for cognitive improvement in this study.

The gut–brain axis is widely recognized as a key modulator of cognitive function, particularly under stress conditions like microgravity [29,30]. Both HU and microgravity are known to cause dysbiosis, characterized by increased pathogenic taxa and decreased beneficial bacteria [31]. Through immune signaling and microbial metabolites production, this imbalance contributes to both systemic and neuroinflammation [32]. As a beneficial bacterium, the proliferation of *Lactobacillus* can reduce intestinal permeability, preventing systemic inflammation caused by the entry of harmful substances into the bloodstream and avoiding the impact of inflammation on the central nervous system. An increased abundance of *Proteus* can disrupt the intestinal mucosal barrier, enabling harmful substances such as lipopolysaccharide (LPS) to enter the bloodstream, disrupt the blood–brain barrier (BBB), and promote neuroinflammation. Together, these microbial shifts can influence cognitive function, providing a mechanistic link between gut ecology and neurocognitive protection. At the genus level, our results showed a lower abundance of *Lactobacillus* in the intestinal tract of HU mice and a higher abundance of *Proteus*, which were consistent with previous publications [33]. Interestingly, PSP treatment effectively restored the diversity and composition of the colon microbiota disrupted by HU. PSP increased the number of beneficial genera like *Lactobacillus* and *Bifidobacterium* that produce SCFAs, including butyrate. SCFAs can cross the blood–brain barrier to influence neurotransmission, while the microbial regulation of tryptophan metabolism impacts serotonin and kynurenine pathways. SCFAs possess neuroprotective properties, including their abilities to enhance neurogenesis, regulate microglial activation, and strengthen the BBB [34,35]. The observed improvement in cognitive functions was probably due to the restoration of these bacteria. Notably, potential biases associated with 16s rRNA sequencing, such as primer amplification efficiency, sequencing depth, and taxonomic database limitations, should be considered when interpreting the microbial community profiles observed in this study. The biological significance of KFGG predictions requires subsequent experimental validation for confirmation.

Moreover, after treatment with PSP, the expression levels of tight junction proteins Claudin 5 and ZO-1 within colon tissue were increased. This strengthens the gut barrier, preventing the translocation of inflammatory mediators that exacerbate neuroinflammation [36]. By reinforcing intestinal integrity, PSP likely interrupts the feed-forward loop of the peripheral inflammation driving central immune activation.

Neuroinflammation is a well-established sign of cognitive impairment caused by microgravity and stress. The NLRP3 inflammasome is a key immune sensor that activates caspase-1 and promotes the release of proinflammatory cytokines IL-1β and IL-18, which can lead to neuronal dysfunctions and memory deficits [37,38]. Similarly, NF-κB signaling pathway is also linked to neurodegeneration and cognitive disorders, which further amplifies inflammatory gene expressions in glial cells [39,40]. Our study found that HU significantly upregulated the expression of NLRP3 and ASC in the hippocampus, along with increasing NF-κB phosphorylation. PSP treatment significantly decreased their expression, suggesting that it inhibits inflammasome activation and the downstream signaling pathway of inflammation. The observed protection of cognitive function is probably mediated through this anti-inflammatory mechanism. These findings align with prior research, showing that natural polysaccharides can attenuate neuroinflammation by blocking NLRP3 inflammasome activation and NF-κB pathways [41]. PSP appears to improve cognitive function by inhibiting these inflammatory pathways. Cleaved caspase-1 (p20 subunit) is the biologically active form responsible for processing pro-IL-1β (precursor interleukin-1β) into its mature cytokine form. Detecting cleaved caspase-1 enables a more precise evaluation of inflammasome activity and its direct impact on IL-1β production. An in-depth exploration of this mechanistic link will be conducted in subsequent studies.

Cognitive dysfunction under microgravity conditions is significantly mediated by neurotransmitter imbalance, specifically Glu and GABA [42]. In this study, PSP treatment restored GABA expression levels in the hippocampus that were significantly decreased by HU and decreased Glu levels. In addition, there is a relationship between inflammatory cytokines and neurotransmitters such as Glu and GABA, which is primarily manifested in the regulatory effects of inflammatory cytokines on neurotransmitter balance. Correlation analysis further supported this link, showing that inflammatory cytokine transcripts (*Il1b* and *Tnf*) were associated with decreased GABA and increased Glu levels in the hippocampus. This suggests that PSP’s anti-inflammatory effects may help restore the excitatory/inhibitory balance, consistent with previous findings that cytokines influence neurotransmitter homeostasis [43]. These findings align with PSP’s reported potential to affect neurotransmitter levels to increase cholinergic transmission and inhibitory signaling, which can improve cognitive functions. Tryptophan (TRP) is not only a vital precursor for the synthesis of 5-HT, but it also plays a pivotal role in the kynurenine (KYN) metabolic pathway [44]. Several metabolites, including KYN, Kynurenic acid (KYNA), and 3-hydroxykynurenine (3-HK), are produced by this pathway. Research has reported that long-term exposure to chronic stress environments leads to the excessive depletion of 5-HT and an imbalance in TRP metabolism, which results in a significant increase in the levels of the key metabolite 3-HK in this pathway [45]. In our study, HU significantly increased kynurenine levels in the hippocampus, reflecting the activation of the kynurenine pathway under stress conditions. This upregulation was accompanied by increased expression levels of NLRP3 and caspase-1, consistent with previous reports that KYN pathway activation can promote inflammasome activity and neuroinflammation [46]. A substantial increase in 3-HK levels suggests that 4 weeks of HU can induce the accumulation of neurotoxic substances and cause brain damage. PSP treatment reversed these changes, reducing kynurenine levels while simultaneously suppressing NLRP3 and caspase-1 expression in the hippocampus. These findings suggest that PSP may exert its neuroprotective effect by modulating tryptophan–kynurenine metabolism and thereby attenuating inflammasome-mediated neuroinflammatory responses in the hippocampus. The results indicated that HU induces cognitive impairment, activates the TRP pathway, and decreases the levels of 5-HT in mice. However, PSP treatment effectively alleviated these alterations. Future research should incorporate markers such as postsynaptic density protein-95 (PSD-95) and brain-derived neurotrophic factor (BDNF) to gain a deeper understanding of the association between neuroinflammation and cognitive outcomes.

The MGB axis plays an integrative role in protecting against cognitive impairment induced by simulated weightlessness. While PSP’s neuroprotective roles in Alzheimer’s disease and stress models have been linked to antioxidative and anti-inflammatory mechanisms, this study provides novel evidence in the specific context of simulated microgravity. PSP acts through multiple interconnected pathways by restoring gut microbiota, inhibiting neuroinflammation via the NLRP3/NK-κB pathway, and regulating neurotransmitter levels. This mechanism of PSP’s action on both gut microbiota and neuroimmune response is supported by other studies that have focused on the MGB axis as an important target for treating cognitive disorders related to environmental stress, including spaceflight [47]. PSP contains polysaccharides with strong antioxidant and immunomodulatory properties, which may suppress oxidative stress and inhibit NF-κB signaling. These properties likely underlie the observed downregulation of NLRP3 inflammasome activation and cytokine release in HU mice. Unlike single-target drugs, PSP works holistically, which reflects the broader therapeutic approach of TCM, and its cognitive effects were proven in an Alzheimer’s disease mouse model [48]. Compared to other natural compounds studied in the HU model, like resveratrol or curcumin, PSP shows comparable or greater potential in improving memory and inhibiting inflammation, like other compounds, including resveratrol [49]. Therefore, combining PSP with such interventions, including structured exercise protocols and pharmacological neuroprotectants, could provide a complementary, multi-target approach to protecting cognitive function under spaceflight conditions. The structural simplicity of key cognitive regions, such as the hippocampus, in the rodent models selected for this study precludes an adequate simulation of the complex neural network activities observed in humans. To further investigate the cognitive impacts of simulated microgravity environments, future research should incorporate validation using more advanced animal models or human trials.

## 5. Conclusions

This study demonstrates for the first time that PSP effectively ameliorates the cognitive impairment induced by HU in mice. The underlying mechanisms may involve restoring the microbiota–metabolite–neurotransmitter axis, along with inhibiting the NF-κB/NLRP3 signaling pathway to attenuate neuroinflammation. These findings open up opportunities for its application in stress-related cognitive disorders and aerospace health but require more investigation for translational and clinical settings.

## Data Availability

The original contributions presented in this study are included in the article/Supplementary Materials. Further inquiries can be directed to the corresponding authors.

## Supplementary Materials

The following supporting information can be downloaded at: https://www.mdpi.com/article/10.3390/nu17193157/s1, Table S1: Primer sequence; Figure S1: Good’s coverage value; Figure S2: Venn diagram based on OUTs; Figure S3: The ratio of Bacteroidetes/Firmicutes; Figure S4: Comparative analysis of LDA scores and functional annotation analysis of KEGG metabolic pathways; Figure S5: Effects of PSP on Neurotransmitter levels in hippocampus, cortex, serum, and colon regions in HU mice.

## Abbreviations

The following abbreviations are used in this manuscript:

HU	Hindlimb unloading
MWMT	Morris water maze test
PAT	Passive avoidance test
NLRP3	Nod-like receptor pyrin domain 3
NF-κB	Nuclear factor kappa-B
ZO-1	Zonula occludens-1
MGB	Microbiota–gut–brain
GABA	Gamma-aminobutyric acid
Glu	Glutamate
3-HK	3-hydroxykynurenine
5-HT	5-hydroxy tryptamine
TRP	Tryptophan
KYNA	Kynurenic acid
Hup	Huperzine A
ASC	Apoptosis-associated speck-like protein
E	Epinephrine
NE	Norepinephrine
