# Supplementary material for: Polygonatum sibiricum Polysaccharides Alleviate Simulated Weightlessness-Induced Cognitive Impairment by Gut Microbiota Modulation and Suppression of NLRP3/NF-κB Pathways
Source: Nutrients. 2025 Oct 5;17(19):3157. doi: 10.3390/nu17193157 (PMC12525828; doi:10.3390/nu17193157)
Supplement: Supplementary file 1 [file nutrients-17-03157-s001.zip › nutrients-3857514-supplementary.pdf]

Supplementary Table S1. Primer sequence

Gene name	Sequence (5′→3′)
Gapdh-F	AACAGCAACTCCCACTCTTC
Gapdh-R	CCTGTTGCTGTAGCCGTATT
Il1b-F	CTCGCAGCAGCACATCAACAAG
Il1b-R	CCACGGGAAAGACACAGGTAGC
Tnf-F	TGTCTACTCCCAGGTTCTCTT
Tnf-R	GCAGAGAGGAGGTTGACTTTC
Tgfb-F	GCTTCTCCCAAGTGTGTCAT
Tgfb-R	GACTGCTGGTGGTGTATTCTT
Arg1-F	TCATGGAAGTGAACCCAACTC
Arg1-R	CGAAGCAAGCCAAGGTTAAAG

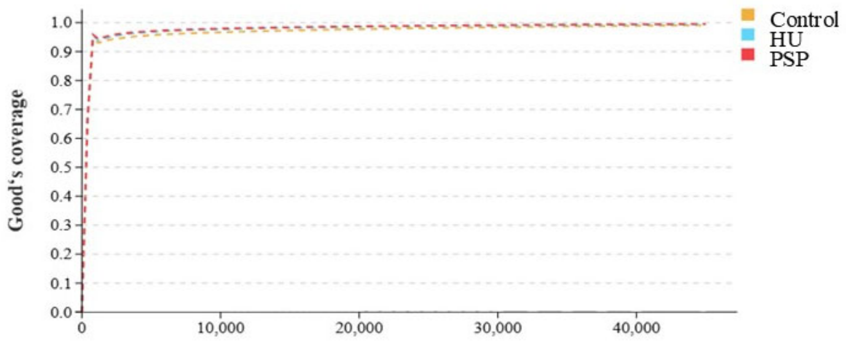

Supplementary Figure S1. Good's coverage value.

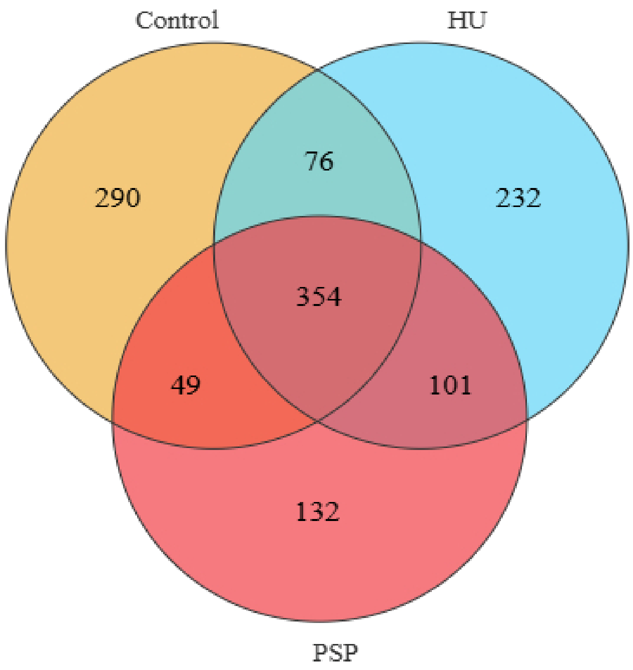

Supplementary Figure S2. Venn diagram based on OUTs.

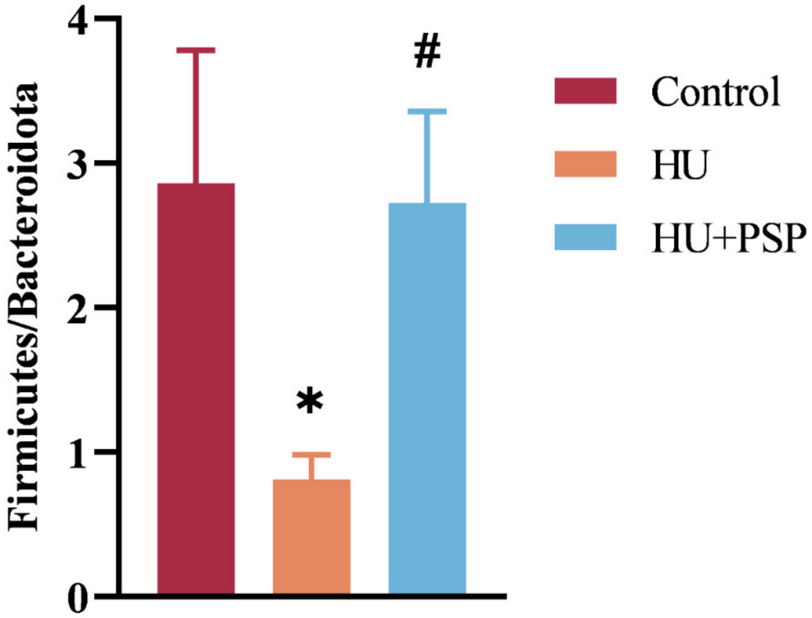

Supplementary Figure S3. The ratio of Bacteroidetes/Firmicutes. (n= 4, mean ± SEM). $^{*}p < 0.05$, vs. Control group. $^{\#}p < 0.05$, versus. HU group.

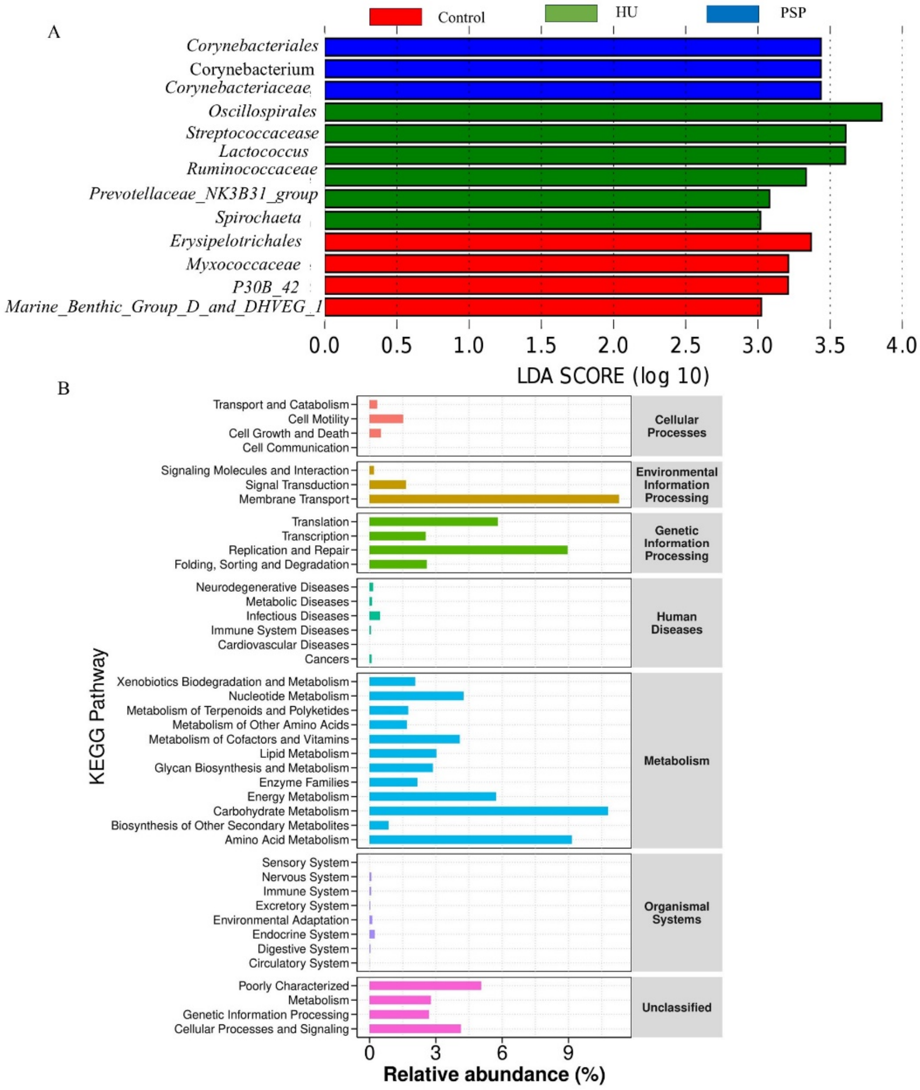

Supplementary Figure S4. Comparative analysis of LDA scores and functional annotation analysis of KEGG metabolic pathways. (A). Distribution histogram of differential bacteria based on LDA Score. (B). KEGG functional pathways following PSP treatment.

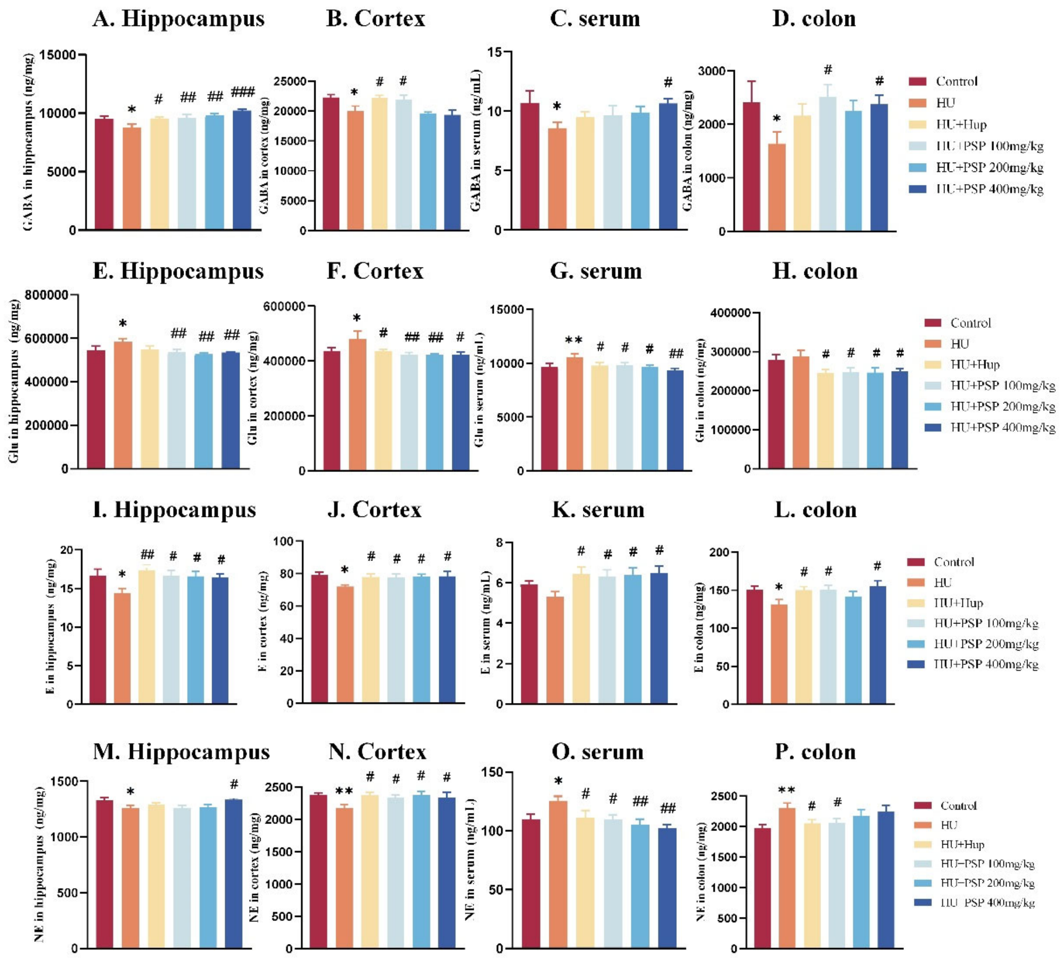

Supplementary Figure S5. Effects of PSP on Neurotransmitter levels in hippocampus, cortex, serum, and colon regions in HU mice. (A-D). Levels of GABA in hippocampus, cortex, serum, and colon; (E-H). Levels of Glu in hippocampus, cortex, serum, and colon; (I-L). Levels of E in hippocampus, cortex, serum, and colon; (M-P). Levels of NE in hippocampus, cortex, serum, and colon. (n=8, mean ± SEM). $^{*}p < 0.05$, $^{**}p < 0.05$ vs. Control group. $^{\#}p < 0.05$, $^{\#\#}p < 0.01$, $^{\#\#\#}p < 0.001$, versus. HU group.
